# Pseudomyogenic hemangioendothelioma of the tibia: a case report

**DOI:** 10.1097/RC9.0000000000000283

**Published:** 2026-02-16

**Authors:** Naoki Takada, Naoto Oebisu, Hana Yao, Kenichi Kohashi, Hidetomi Terai

**Affiliations:** a Department of Orthopedic Surgery, Graduate School of Medicine, Osaka Metropolitan University, Osaka, Japan; b Department of Pathology, Graduate School of Medicine, Osaka Metropolitan University, Osaka, Japan

**Keywords:** bone tumor, case report, FOSB, pseudomyogenic hemangioendothelioma, rare endothelial neoplasm

## Abstract

**Introduction and importance:**

Pseudomyogenic hemangioendothelioma (PMHE) is a rarely metastasizing intermediate endothelial neoplasm. It typically presents as multiple, discontinuous nodules in the lower extremities of young adult males. Most cases present skin or soft-tissue tumors, but intraosseous lesions without soft-tissue involvement are extremely rare. We report a case of PMHE of the tibia, diagnosed with FOSB staining.

**Presentation of case:**

A 36-year-old man presented with pain in the lower left leg after exercise and at night. X-ray, SPECT/CT, and MRI revealed two discontinuous lytic lesions within the cortical bone of the left tibia. A curettage procedure was performed for biopsy, but a diagnosis could not be made for a long time. After further consultation, FOSB staining was positive in the nucleus, leading to a definitive diagnosis of PMHE. No recurrence or metastasis was observed 2 years after surgery.

**Clinical discussion:**

PMHE is a rare endothelial tumor, and cases limited to bone without soft tissue involvement are exceptional. Diagnosis is difficult because of overlap with myogenic and epithelioid tumors, but nuclear FOSB staining is a reliable marker. Although local recurrence is frequent, metastasis is rare, underscoring the importance of accurate diagnosis and long-term follow-up.

**Conclusion:**

This case highlights the importance of considering PMHE in the differential diagnosis of bone tumors, the utility of FOSB immunostaining in confirming the diagnosis, and the need for long-term follow-up given the high risk of local recurrence.

## Introduction

Pseudomyogenic hemangioendothelioma (PMHE) is a rare, intermediate endothelial neoplasm that may rarely metastasize, primarily affecting young adult males. It most commonly originates in the lower extremities, followed by the upper extremities and trunk, often presenting as multiple, discontinuous nodules^[1]^. Many cases involve skin and soft tissue lesions, and osteolytic lesions may occasionally be present; however, cases with bone lesions without soft tissue involvement are extremely rare^[2]^. Pathologically, it resembles myogenic tumors or epithelioid sarcomas^[1]^, but FOSB positivity in the nucleus is a consistent finding and serves as an important diagnostic clue^[3]^. In the following report, we present a case in which PMHE was definitively diagnosed based on FOSB staining after tumor resection for multiple osteolytic lesions of the tibia.

**HIGHLIGHTS**

- Pseudomyogenic hemangioendothelioma (PMHE) is a rare intermediate endothelial tumor.
- Purely intraosseous PMHE without soft tissue involvement is extremely uncommon.
- Local recurrence is frequent, but metastasis is rare and usually delayed.
- This case demonstrates the diagnostic importance of FOSB positivity in the differential diagnosis of multiple osteolytic lesions.

## Case report

A 36-year-old man presented with pain in the lower left leg, occurring after exercise and at night. He had no relevant medical or family history.

On clinical examination, there was pain in the left lower leg but no local swelling or tenderness and no functional impairment of the limb.

**Figure 1.**
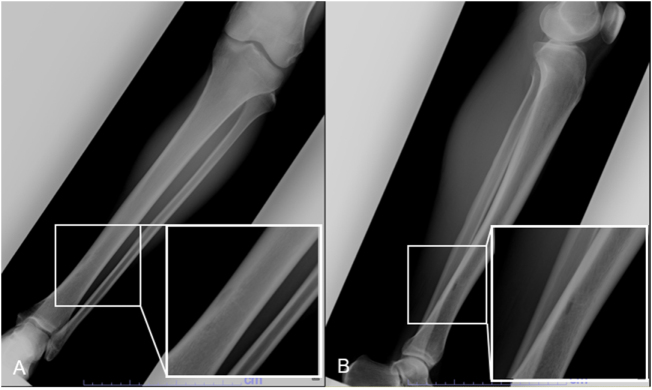
Anteroposterior (A) and mediolateral (B) radiograph showing discontinuous osteolytic lesions in the tibia.

**Figure 2.**
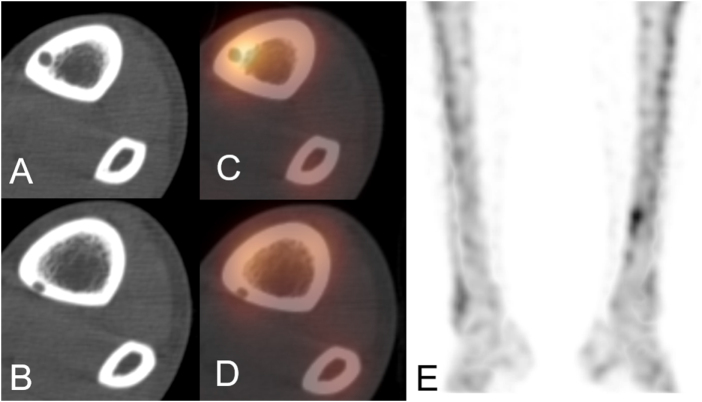
CT images (A and B), CT/SPECT images (C and D), and bone scintigraphy images (E) demonstrating intracortical lesions and abnormal uptake.

**Figure 3.**
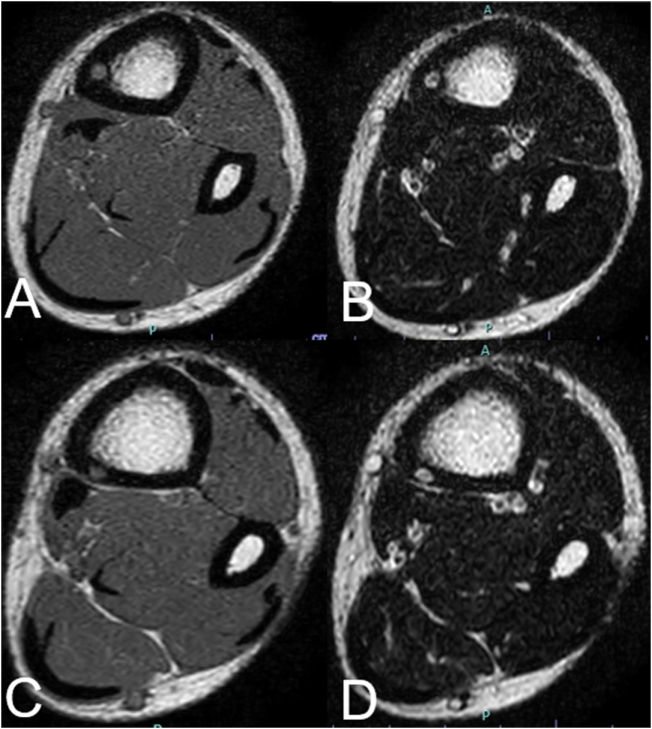
MRI showing isointense on T1-weighted images (A and C) and hyperintense on T2-weighted images (B and D).

Imaging evaluation revealed two discontinuous osteolytic changes in the distal shaft of the left tibia on X-ray (Fig. 1). CT demonstrated two additional cortical lesions, each less than 1 cm, at separate sites. SPECT/CT and bone scintigraphy showed abnormal radiotracer uptake corresponding to these areas (Fig. 2). On MRI, the lesions appeared in innocently on T1-weighted images and hyperintense on T2-weighted images without surrounding bone marrow edema (Fig. 3).

Laboratory investigations, including inflammatory markers and tumor markers, were within normal limits.

The presence of multiple well-defined osteolytic cortical lesions is extremely rare, and diagnosis based on imaging alone was challenging. Differential diagnoses considered included metastatic bone tumors, osteomyelitis, osteofibrous dysplasia, and osteoid osteoma, but none could be confirmed without histopathological assessment.

**Figure 4.**
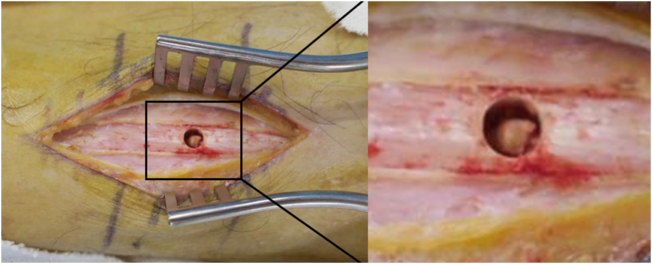
Gross surgical findings.

**Figure 5.**
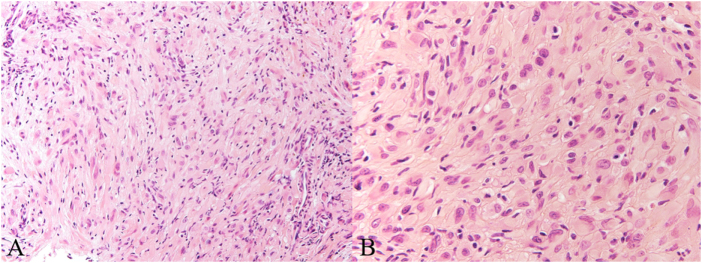
H&E staining revealed tumor cells exhibiting loose bundles or disorganized proliferation patterns (A), along with epithelioid cells possessing distinct nucleoli (B).

**Figure 6.**
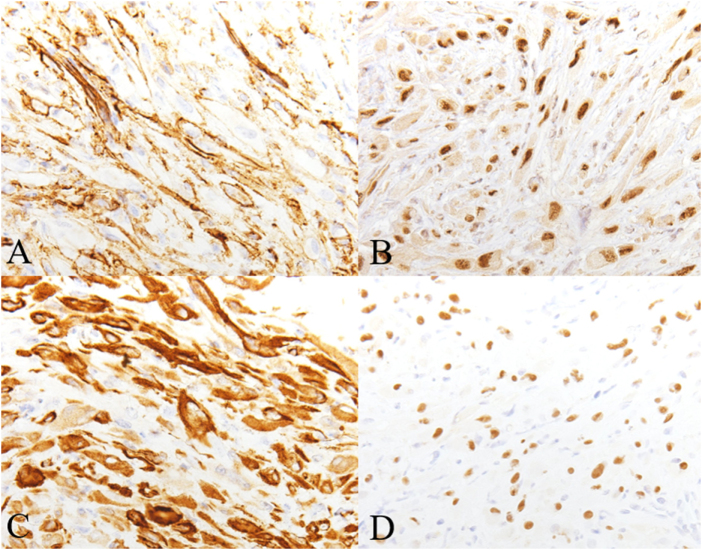
The tumor cells typically express CD31 (A), ERG (B), AE1/AE3 (C), and FOSB (D).

No specific pre-operative optimization was required beyond standard assessment and fasting instructions. Biopsy, curettage, and artificial bone grafting were performed on two lesions (Fig. 4). The operations were performed by a dedicated orthopedic oncology specialist at Osaka Metropolitan University Hospital, a tertiary referral center for musculoskeletal tumors. There were no deviations from the initial management plan, and all interventions proceeded as scheduled. The initial pathological diagnosis of the surgical specimen revealed atypical cells proliferating in sheet- or cord-like patterns, with nuclei that were round or irregular in shape and contained abundant eosinophilic cytoplasm. Mitotic figures were sparsely observed. No significant nuclear atypia or necrosis was noted. The stroma was fibrous or partially myxoid (Fig. 5). Immunohistochemical staining showed AE1/3 (+), CD31 (+), ERG (+) (Fig. 6), CD34 (−), CD68 (−), α-SMA (−), S100 (−), and Ki-67 LI approximately 1.1%. Based on these findings, the diagnosis was epithelioid hemangioendothelioma.

When pathological diagnosis was requested again 1 year after surgery, FOSB positivity was confirmed, and the diagnosis was revised to PMHE (Fig. 6). During the 2-year follow-up period after surgery, no local recurrence was detected on serial X-rays of the left tibia, and no distant metastases, including lung involvement, were identified on chest CT scans. The patient remained free of pain and maintained full function of the lower limb. No adverse events or complications were observed throughout the follow-up.

## Discussion

PMHE is a rare endothelial neoplasm, first described by Mirra in 1992^[3]^, and is categorized in the WHO classification of soft tissue tumors as an intermediate endothelial tumor with distinctive histological and molecular characteristics^[4]^. Clinical symptoms vary, with approximately half of cases being painless and the other half painful^[1]^. Most lesions measure 1–2.5 cm in diameter, with only 10% exceeding 3 cm. PMHE typically involves the skin and subcutaneous tissue (75%), muscle (50%), and bone (20%)^[5]^. However, PMHE presenting exclusively with intraosseous lesions, without associated soft tissue disease as in the present case, is extremely rare. To date, only 14 such cases have been reported in the literature^[2,6–8]^ (Table1).

**Table 1:** Previous reports of PMHE involving bone lesions only, without accompanying soft tissue lesions

Study (Ref)	Age/sex	Tumor site	Diagnostic method	Primary treatment	Outcome of surgery site	Outcome of ovservation site	Remarks
Inyang A *et al*, 2016^[2]^	12-74/M(9)F(1)	Lower extremity (45%)/spine and pelvis (25%)/upper extremity (15%)	Biopsy (9), surgical pathology (1)	Resection (4), curettage (1), observation (5)	Stable (4), reccurence (1)	Stable (4), Progressing (5), DOOD(1)	Finally amputation needed (1)
Friel NA *et al*, 2014^[6]^	8/F	Proximal femur	Open biopsy	Wide resection	Reccurence (-), new lesion (-)	No observation	
Righi A *et al*, 2014^[7]^	25/M, 66/F	Radius, tibia, fibula and almost all bones of the foot	Open biopsy (2)	Resection, curretage	Reccurence (1), new lesion (1)	Stable (1)	Finally amputation needed (1)
McGinity M *et al*, 2013^[8]^	25/M	The third and fourth thoracic vertebrae	Open biopsy → Surgical pathology	Gross total resection	Reccurence (+), new lesion (+)		

PMHE frequently recurs, with many patients’ requiring repeat surgery, and in two reported cases, amputation was necessary. The tumorigenesis was initially linked to a balanced translocation t(7;19)(q22;q13), resulting in a SERPINE1–FOSB fusion^[9,10]^. More recently, the ACTB–FOSB fusion gene has been identified in approximately half of cases^[11]^. Histopathologically, PMHE is characterized by sheets or fascicles of plump spindle cells with abundant eosinophilic cytoplasm. These cells may resemble rhabdomyoblasts, and occasional epithelioid cells can mimic myogenic tumors or epithelioid sarcomas. In intraosseous cases, epithelioid cells with eosinophilic cytoplasm tend to predominate. Most tumors show only mild atypia and few mitotic figures, although about 10% demonstrate marked pleomorphism and prominent nucleoli^[1]^.

Immunohistochemically, PMHE often shows diffuse positivity for keratin (AE1/AE3) and endothelial transcription factors such as FLI1 and ERG. CD31 is positive in roughly half of cases and αSMA in one-third. Nuclear FOSB expression is the most consistent and diagnostically important finding^[3]^. In the present case, strong FOSB positivity was a major factor in reaching the diagnosis.

The relationship between surgical margin status and recurrence remains unclear, and there is no consensus regarding the optimal margin. Approximately 60% of cases develop local recurrence or new lesions at adjacent sites, whereas lymph node or distant metastases are rare (<5%) and typically occur years or decades later, most commonly in the lungs, bones, or soft tissue^[1]^. In our case, curettage was performed, and no recurrence or metastasis has been observed during the 2-year follow-up.Recent literature, including the systematic review by Brance *et al* has reported cases involving both bone and soft-tissue components and highlighted variable therapeutic responses, such as favorable outcomes with pamidronate^[12]^. These findings suggest that PMHE exhibits heterogeneous clinical behavior and may require individualized management strategies. Incorporating this broader context reinforces the importance of accurate diagnosis and long-term surveillance, particularly in cases with purely intraosseous presentation such as the present case.

## Strengths and limitations

This case illustrates the rare occurrence of PMHE confined to bone, highlighting the diagnostic value of FOSB immunostaining with relevance for orthopedic oncology, radiology, and pathology. However, the relatively short follow-up period of 2 years and the use of curettage rather than wide resection limit the assessment of long-term disease control. Continued surveillance and accumulation of further cases are needed to better define the prognosis and optimal management strategy.

## Conclusion

This case highlights an unusual presentation of PMHE limited to bone, without concomitant soft tissue lesions. PMHE should be considered in the differential diagnosis when multiple, non-continuous osteolytic lesions are identified. Nuclear FOSB immunostaining is of high diagnostic value, and long-term follow-up is essential due to the risk of local recurrence.

## Patient perspective

The patient expressed relief at obtaining a clear diagnosis and satisfaction with the treatment outcome. He recognized the need for long-term follow-up and was willing to comply with regular surveillance.

## Data Availability

Data supporting this study are available from the corresponding author upon reasonable request. No additional datasets were generated or analyzed.

## Reporting guidelines

This case report has been reported in line with the SCARE 2025 criteria^[13]^.

## References

[1] Hornick JL Fletcher CDM. Pseudomyogenic hemangioendothelioma: a distinctive, often multicentric tumor with indolent behavior. Am J Surg Pathol 2011;35:190–201. 21263239 10.1097/PAS.0b013e3181ff0901

[2] Inyang A Mertens F Puls F. Primary pseudomyogenic hemangioendothelioma of bone. Am J Surg Pathol 2016;40:587–98. 26872012 10.1097/PAS.0000000000000613

[3] Mirra JM Kessler S Bhuta S. The fibroma-like variant of epithelioid sarcoma: a fibro-histiocytic/myoid cell lesion often confused with benign and malignant spindle cell tumors. Cancer 1992;69:1382–95. 1371711 10.1002/1097-0142(19920315)69:6<1382::aid-cncr2820690614>3.0.co;2-y

[4] Jo VY Fletcher CD. WHO classification of soft tissue tumours: an update based on the 2013 (4th) edition. Pathology 2014;46:95–104. 24378391 10.1097/PAT.0000000000000050

[5] Amary MF O’Donnell P Berisha F. Pseudomyogenic (epithelioid sarcoma-like) hemangioendothelioma: characterization of five cases. Skeletal Radiol 2013;42:947–57. 23381465 10.1007/s00256-013-1577-8

[6] Friel NA Rothenberg AC Weiss K. Pseudomyogenic hemangioendothelioma of bone initially managed as slipped capital femoral epiphysis: a case report. J Cancer Ther 2014;5:363–68.

[7] Righi A Gambarotti M Picci P. Primary pseudomyogenic hemangioendothelioma of bone: report of two cases. Skeletal Radiol 2015;44:727–31. 25300339 10.1007/s00256-014-2024-1

[8] McGinity M Bartanusz V Dengler B. Pseudomyogenic hemangioendothelioma (epithelioid sarcoma-like hemangioendothelioma, fibroma-like variant of epithelioid sarcoma) of the thoracic spine, Eur. Spine J 2013;22:S506–11. 10.1007/s00586-013-2727-3 PMC3641265 23435749

[9] Trombetta D Magnusson L von Steyern FV. Translocation t(7;19)(q22;q13)−a recurrent chromosome aberration in pseudomyogenic hemangioendothelioma? Cancer Genet 2011;204:211–15. 21536240 10.1016/j.cancergen.2011.01.002

[10] Walther C Tayebwa J Lilljebjörn H. A novel SERPINE1-FOSB fusion gene results in transcriptional up-regulation of FOSB in pseudomyogenic hemangioendothelioma. J Pathol 2014;232:534–40. 24374978 10.1002/path.4322

[11] Agaram NP Zhang L Cotzia P. Expanding the spectrum of genetic alterations in pseudomyogenic hemangioendothelioma with recurrent novel ACTB-FOSB gene fusions. Am J Surg Pathol 2018;42:1653–61. 30256258 10.1097/PAS.0000000000001147 PMC6608746

[12] Brance ML Cóccaro NM Roitman P. Pseudomyogenic hemangioendothelioma with bone and soft tissue involvement with favorable response to pamidronate: a case report and systematic review of the literature, Arch. Osteoporos 2022;17:28. 10.1007/s11657-022-01062-4 35106633

[13] Kerwan A Al-Jabir A Mathew G. Revised Surgical CAse REport (SCARE) guideline: An update for the age of Artificial Intelligence. Prem J Sci 2025;10:100079.

